# Simulation of the mechanical interlocking capacity of a rough bone implant surface during healing

**DOI:** 10.1186/s12938-015-0038-0

**Published:** 2015-05-21

**Authors:** Anders Halldin, Mats Ander, Magnus Jacobsson, Stig Hansson

**Affiliations:** Department of Prosthodontics, Faculty of Odontology, Malmö University, 205 06 Malmö, Sweden; DENTSPLY Implants, Box 14, 43121 Mölndal, Sweden; Department of Applied Mechanics, Chalmers University of Technology, 41296 Gothenburg, Sweden

**Keywords:** Bone-to-implant interface shear strength, Biomechanics, Bone healing, FEA

## Abstract

**Background:**

When an implant is inserted in the bone the healing process starts to osseointegrate the implant by creating new bone that interlocks with the implant. Biomechanical interlocking capacity is commonly evaluated in *in vivo* experiments. It would be beneficial to find a numerical method to evaluate the interlocking capacity of different surface structures with bone. In the present study, the theoretical interlocking capacity of three different surfaces after different healing times was evaluated by the means of explicit finite element analysis.

**Methods:**

The surface topographies of the three surfaces were measured with interferometry and were used to construct a 3D bone-implant model. The implant was subjected to a displacement until failure of the bone-to-implant interface and the maximum force represents the interlocking capacity.

**Results:**

The simulated ratios (test/control) seem to agree with the *in vivo* ratios of Halldin et al. for longer healing times. However the absolute removal torque values are underestimated and do not reach the biomechanical performance found in the study by Halldin et al. which might be a result of unknown mechanical properties of the interface.

**Conclusion:**

Finite element analysis is a promising method that might be used prior to an *in vivo* study to compare the load bearing capacity of the bone-to-implant interface of two surface topographies at longer healing times.

## Background

When an implant is inserted into bone the healing process starts by creating new bone that interlocks with the implant surface, so called osseointegration. It has been found that an implant with a rough surface topography enhances osseointegration and load bearing capacity [1, 2]. A significant amount of research has been undertaken in order to identify the biomechanical response to a structured surface, which is characterized by a set of statistical surface roughness parameters [3–7]. The general result of *in vivo* experiments is that increased implant surface roughness (S_a_ value) of cylindrical implants results in increased interfacial shear strength [8]. Whether this empirical correlation is an effect of enhanced mechanical strength of the bone caused by the biological response to the surface and/or enhanced interlocking capacity is unclear. In addition, it is unclear whether surface roughness parameters truly reflect the load bearing capacity [9]. Hansson and Norton [10] developed artificial surfaces characterized by various pit size, pit shape and pit density which were used to simulate the theoretical interfacial load bearing capacity. They found that the topographical structure affects the interfacial load bearing capacity [10]. However, the theoretical surfaces considered by Hansson and Hansson [9] do not represent the surface topography of implants. Besides the implant surface topographical structure, the interfacial load bearing capacity depends on the mechanical properties of the bone during healing. In brief, bone formation in a gap proceeds, regardless of the presence of an implant, in two steps: (1) woven bone formation (2) remodeling of woven bone to lamellar/Harversian bone [11, 12]. Woven bone is characterized by a random arrangement of collagen fibers with poor mechanical properties [13]. Woven bone is thereafter remodeled to Haversian bone over time by basic multicellular units (BMU) [14]. The mineralization process of a new Haversian system can be divided into two stages; primary mineralization and secondary mineralization [15]. Primary mineralization is characterized by a rapid, constant rate of mineralization that proceeds until 50–60% of the mineralization maximum has been reached [15, 16]. Following primary mineralization, a decreased rate of mineralization progressively continues and typically stabilizes at around 90–95% of the maximum level [15, 16]. The current knowledge of the mechanical properties of bone during healing is scarce. However, it is known that the degree of mineralization greatly affects the mechanical properties of bone [17–20]. It is evident that newly formed bone has different mechanical properties compared to mature bone due to different degrees of mineralization [21, 22]. Nano indentation has been used to investigate the mechanical properties of newly formed bone. Leong and Morgan [13] measured the mechanical properties of fractured rat bones after 24 days of healing and found that Young’s modulus of woven and cortical bone was 36.2 MPa and 7.2 GPa respectively. In a study by Ishimoto et al. [23] the mechanical properties of regenerated (2 weeks) and mature rabbit bone were measured. They obtained an elastic modulus of 17.0 and 27.6 GPa respectively which is higher than the maximum Young’s modulus for mature rabbit bone (~8 GPa) found by Isaksson et al. [21]. Few studies have measured the mechanical properties of bone in the vicinity of implants. Chang et al. [24] obtained a value of Young’s modulus of pig alveolar bone close to an implant surface, after one month of healing, of 6.17 GPa that gradually increased to 7.9 GPa at a distance of 150 µm from the implant surface. Finally, it reached 10.1 GPa at a distance of 1,500 µm from the implant surface. This is slightly lower than what Vayron et al. [25] found in rabbit bone after longer healing times (7 and 13 weeks). An alternative way to determine the mechanical properties during healing was used in a study by Warzen et al. [26]. They measured the stiffness of the implant bone interface *in vivo* of mice hind legs after 0–6 days of healing and by reverse engineering, obtained values of Young’s modulus ranging from 3 to 17 MPa. The mechanical properties of the bone and the surface topography affect the bone implant interfacial shear strength [10]. The objective of the present study was to estimate the theoretical bone-to-implant interfacial shear strength for different surface topographies during healing by means of finite element analysis (FEA).

## Methods

### Geometrical representation of a surface topography

**Figure 1:**
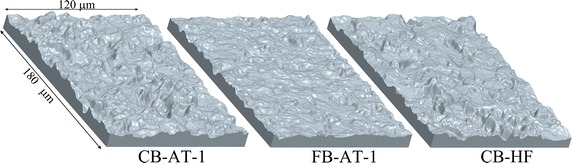
Surface geometry. Geometrical representation of the selected patch of the implant surfaces (CB-AT-1, FB-AT-1 and CB-HF) derived from interferometry measurements in the study by Halldin et al. [27]. Surface characteristics of the patches are presented in Table 3.

The coordinates (x, y and z) from a representative patch of each of the three different (A, B and C) surfaces used in the study by Halldin et al. [27] were extracted from interferometry measurements. Group A were blasted with coarse titanium particles thereafter treated in oxalic acid and hydrofluoric acid sequentially (CB-AT-I). Group B were blasted with fine particles titanium oxide particles and thereafter treated in oxalic acid and hydrofluoric acid sequentially (FB-AT-I). Group C implants were blasted with coarse titanium particles thereafter treated in hydrofluoric acid (CB-HF). The representative patches were selected to have surface average height values (S_a_) close to the mean S_a_ values of the implant in Halldin et al. [27]. The x, y and z coordinates were imported into MATLAB (MATLAB 2013b, Mathworks Inc., USA) to calculate the average height values (S_a_ [μm]), root-mean-square (S_q_ [μm]), skewness (S_sk_ [μm/μm]) and the root-mean-square of the surface slope (S_dq_ [μm/μm]) of the selected patches [8]. Thereafter, the surfaces were exported to STL file format followed by conversion with high accuracy, to IGES format using Geomagic studio® (Geomagic Solutions, Cary, NC, USA). The IGES surfaces were imported into Pro Engineer (PTC, Needham, MA, USA) for creation of a 3D geometry (Figure 1). Finally the geometries were imported to ANSYS® 14.5 (ANSYS INC, Canonsburg, PA, USA) for simulation of the interfacial load bearing capacity.

### Finite element model

**Figure 2:**
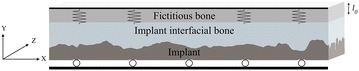
Illustration of the 3D FEA model with boundary conditions.

The bone-to-implant interface was modeled according to Figure 2. The implant surface was in contact with interfacial bone which in turn was adjacent to the fictitious bone. The fictitious bone represents the structural stiffness of the surrounding bone and was modeled as an isotropic material with a thickness (*l*_0_) of 10 µm and a Young’s modulus represented by E_s_. Substituting the surrounding bone by a fictitious bone results in a less computationally heavy model. The implant was locked in y direction and was moved in x direction by a constant acceleration (a_x_) during the simulation time. The interface between interfacial bone and fictitious bone was fixed in x direction and free to move in y direction. The outer sides of the X–Y plane of the 3D model were restricted to move in z direction. The implant was modeled as a rigid component and the interface bone-to-implant interface was modeled as a frictional contact with an assumed coefficient of friction of 0.4 [28].

### Mechanical properties

The Young’s modulus (E_s_) of the fictitious bone was determined by simulating the deformation (*d*) at a predefined internal pressure (*P*) of 100 MPa in a 2D plane strain model representing a disc of mature bone. The disc had an inner diameter of 3.5 mm similar to the outer diameter of the implant in the study by Halldin et al. [27]. The structural stiffness was computed for different outer diameters (D_o_) of the disc ranging from 2.5 to 12.5 mm. The fictitious Young’s modulus E_s_, for each outer diameter (D_o_) with corresponding deformation (d), was calculated using Eq. (1).

1 $$E_{s} = \frac{{P \cdot l_{0} }}{d}$$

**Figure 3:**
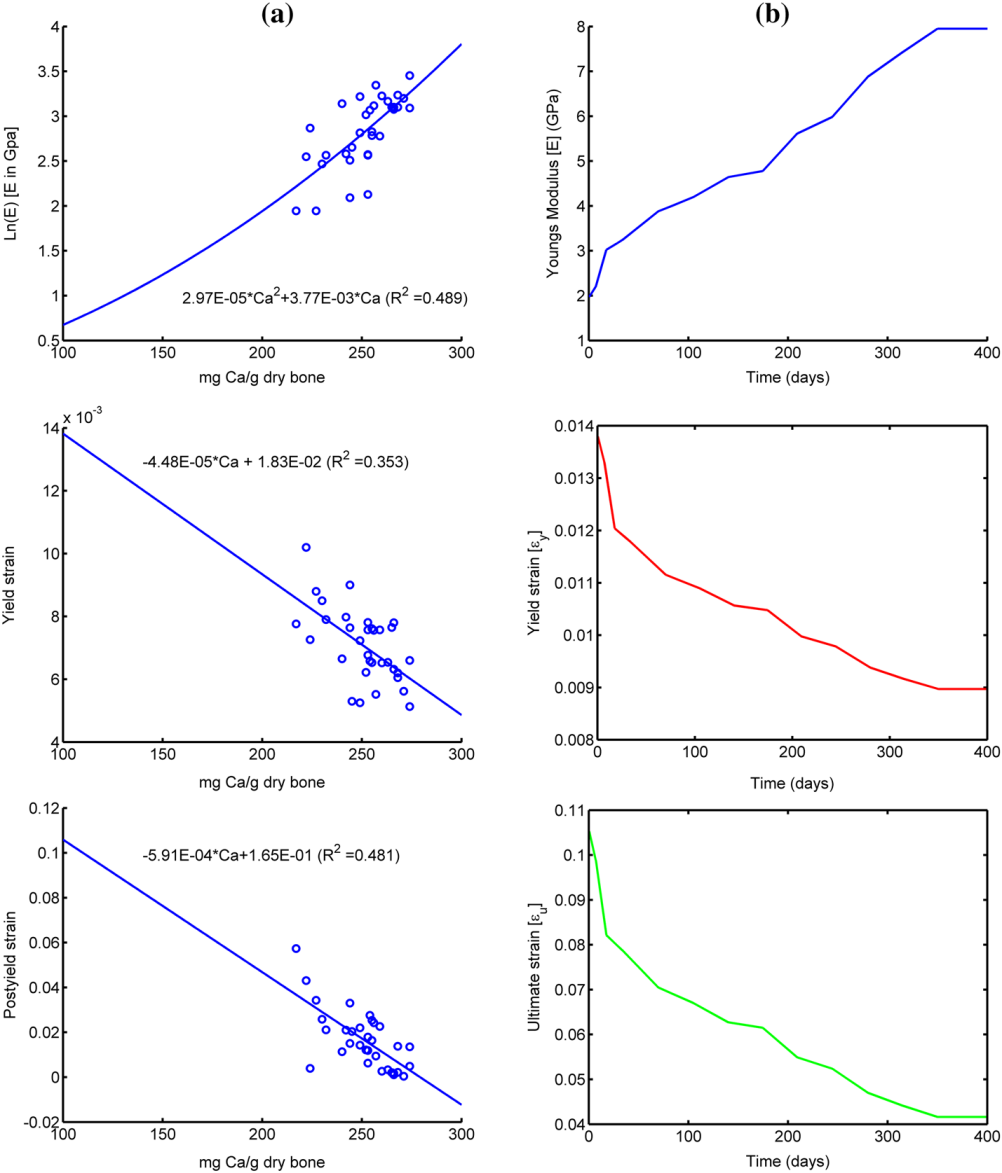
Estimation of mechanical properties during healing: **a** Derived functions (*solid line*) for correlation between the mineral content and the mechanical properties derived from the data (o) published by Currey [17]. **b** Mechanical properties during healing derived from the data presented by Currey [17] and Fuchs et al. [15].

**Figure 4:**
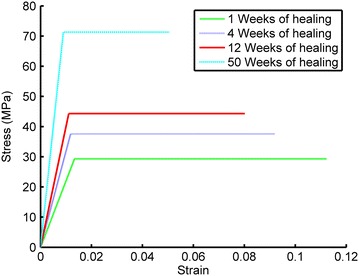
Stress–strain curves at different healing times.

Since the current knowledge of the mechanical properties of bone in the vicinity of an implant during healing is limited, the assumed mechanical properties of bone during healing were derived from the correlation between mechanical properties and mineral content [17] and from the correlation between mineral content and healing time [15]. Currey [17] investigated the mineral content, expressed as milligrams of calcium (Ca) per gram dried defatted bone, for various species with corresponding Young’s modulus (E), yield strain (ε_y_) and post yield strain (ε_py_). Using Currey´s data the correlation between Ca and E, ε_y_ and ε_py_ can be identified (Figure 3a). Furthermore, by assuming 100% mineralization after 350 days of healing [15], and a corresponding Young’s modulus of 7,950 MPa [21], the Young’s modulus (E), yield strain (ε_y_) and ultimate strain (ε_u_ = ε_y_ + ε_py_) at different healing times can be approximated (Figure 3b). In the present study it was assumed that the implant interfacial bone exhibits a bilinear isotropic material behavior without hardening. Thus the stress strain curves and the mechanical properties at different healing times can be estimated (Figure 4; Table 1). The strength and Young’s modulus of titanium are an order of magnitude higher than those of the bone. Therefore, to reduce computing time the implant was assumed to be rigid. In the present study the shear strength of a bone-to-implant interface was simulated with explicit finite element analysis (Figure 2) using the ultimate strain as a failure criterion. According to the simulations the maximum shear force occurred at implant displacements of less than 0.75 µm. Therefore, the simulations were discontinued after an implant displacement of 0.75 µm was reached. The displacement (0.75 µm) was achieved by a constant acceleration of the implant during the simulation time. Using the representative patch area the maximum reaction force between implant interfacial bone and the fictitious bone was converted, to interfacial shear strength (τ_p_). In explicit FE simulation the computing time depends on the choice of simulation time, minimum element size and material density [29]. Reduced computing time is obtained by decreased simulation time (increased acceleration), increased material density and increased minimum element size [29]. Prior to the 3D simulation a parameter study (convergence test) with several combinations of different values of the acceleration (P1), material density increase (P2) and minimum element size (P3) (Table 2) was performed to obtain reasonable computing time without compromising the accuracy. The parameter study was performed by simulating the reaction force on a 2D profile. The 2D profile was extracted from an x–y plane of the 3D model (Figure 2). The meshes were auto generated in ANSYS® with different values of the minimum element size at the bone-to-implant interface. Otherwise the simulations were performed with the same settings. The simulated shear strength for the three surfaces were converted to removal torque value (RTQ) of an implant with the same design as in the Halldin et al. [27] study according to Eq. (2).

2 $$RTQ = \tau_{p} \cdot A_{i} \cdot r \cdot BIC$$

where *A*_*i*_ represents the outer threaded area of the implant which was 78 mm^2^, *r* represents the radius to the implant surface, which was 1.75 mm, and *BIC* represents the bone to implant contact obtained in the *in vivo* study [27], which was set to 40% for all three surfaces.

**Table 1:** Mechanical properties during healing

Healing time (weeks)	E (GPa)	σ_y_ (MPa)	ε_u_ (%)
1	2,200	29.3	11.22
4	3,164	37.6	9.2
12	4,009	44.3	8.0
50	7,950	71.3	5.1

The properties were derived from the data by Currey [17] and Fuchs et al. [15].

**Table 2:** Convergence analysis

P1	P2	P3
Acceleration m/s^2^	Material density increase factor	Minimum element size (µm)
6	1	0.5
66.7	100	0.625
600	1,000	0.75
60,000	10,000	0.875
	1,000,000	1.0

Values used in the 2D analysis to identify appropriate parameter settings to obtain reasonable computing times for the following 3D simulations.

## Results

**Figure 5:**
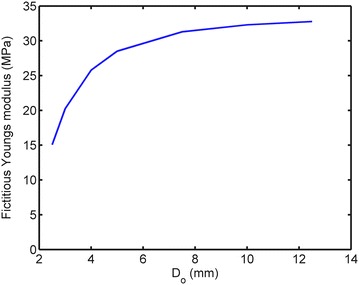
Calculation of fictitious Young’s modulus. Young’s modulus of mature bone surrounding a 3.5 mm implant with different values of the outer diameter (D_0_).

**Figure 6:**
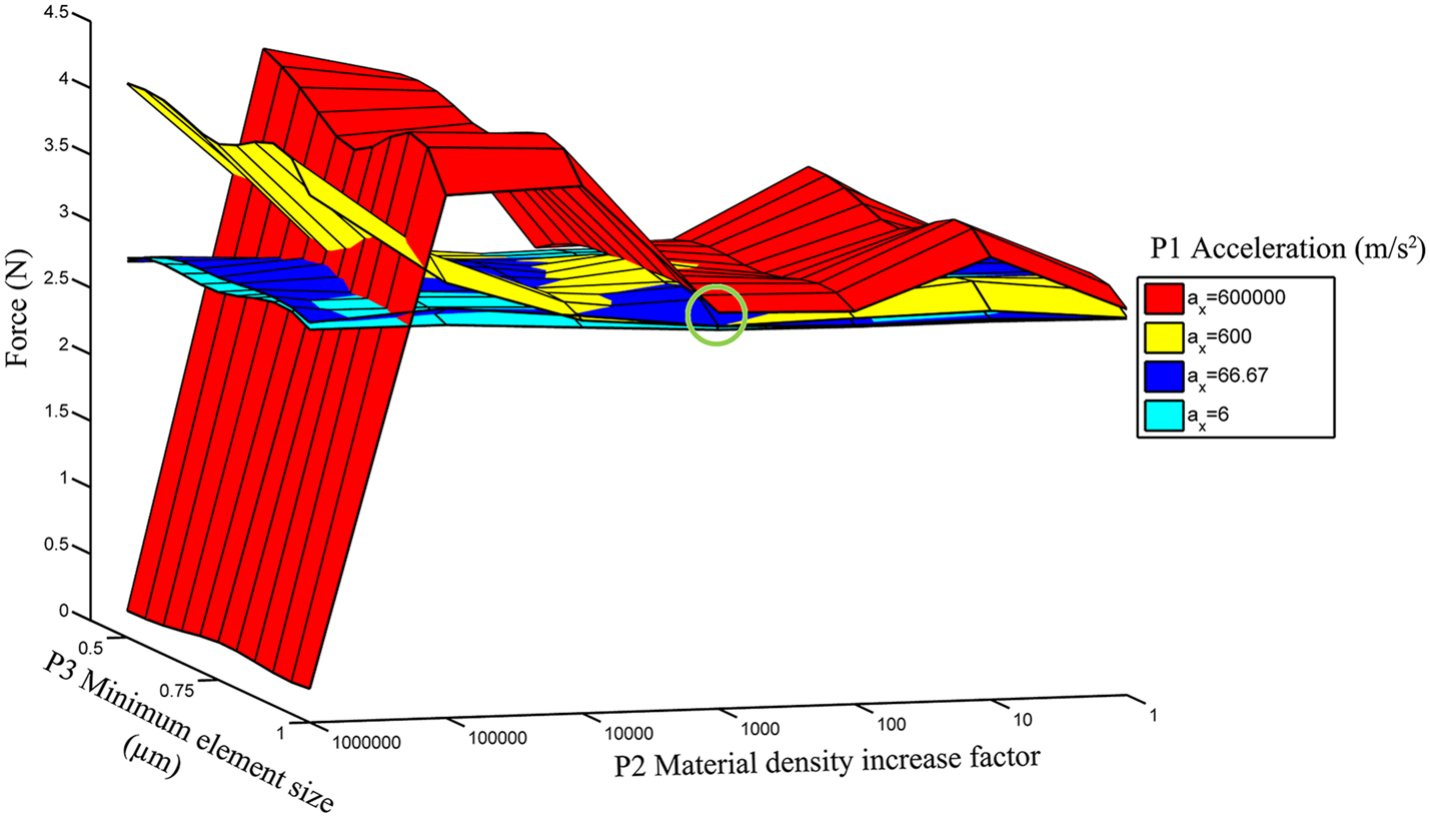
Convergence test analysis. The results of the 2D simulation indicate that a parameter setting of 600 m/s^2^, material density increase of 1,000 and minimum element size of 1 µm (*green circle*) do not significantly affect the reaction force compared to lower parameter values.

**Figure 7:**
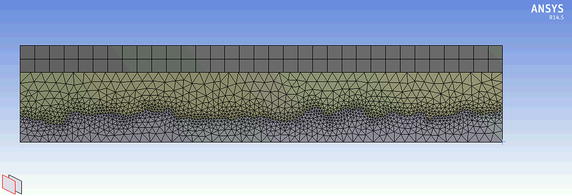
Illustration of mesh. Mesh used in the present simulation with a minimum element size of 1 µm.

**Figure 8:**
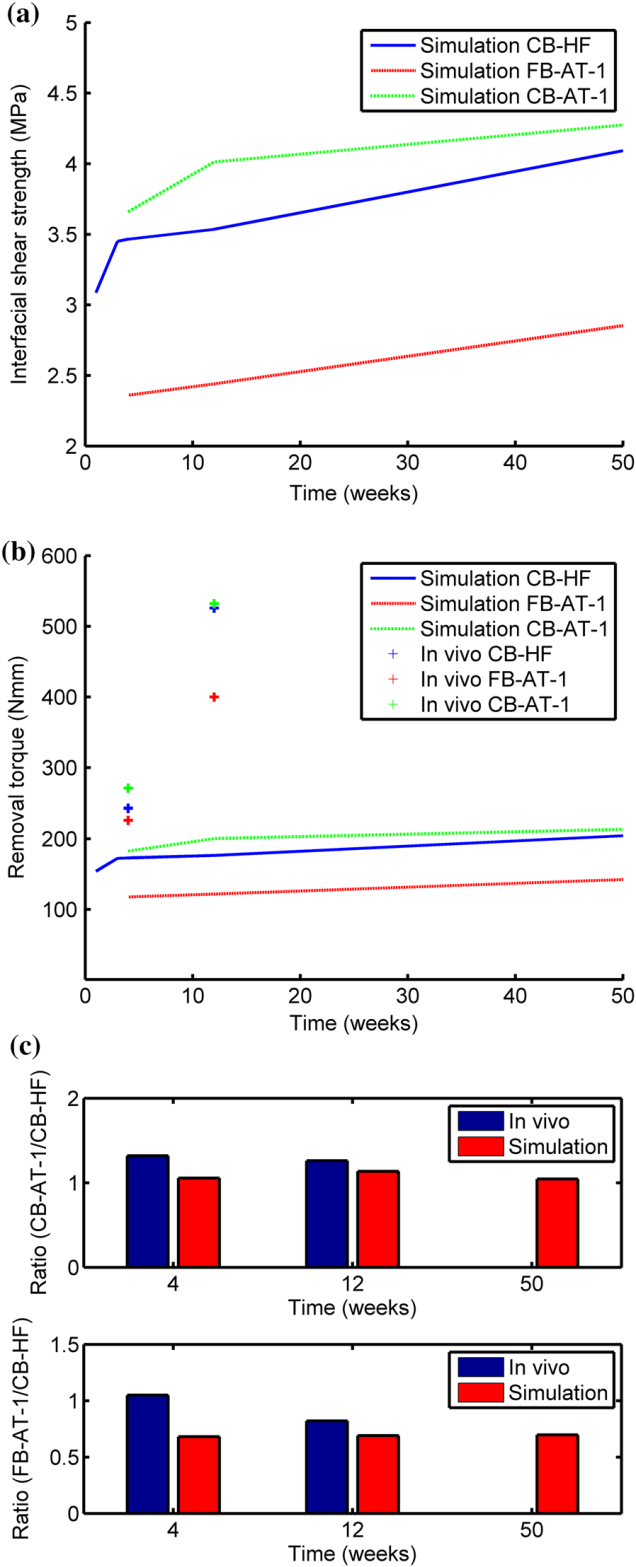
Load bearing capacity. **a** Maximum interfacial shear strength of the three surfaces simulated in the 3D FEA model. **b** Theoretical removal torque calculated according to Eq. (2) compared to the mean removal torque value found by Halldin et al. [27]. **c** The ratio (test/control) of the simulated interfacial shear strength and the mean of the corresponding removal torque ratios of the *in vivo* study by Halldin et al. [27].

The surface roughness (S_a_ value) of the selected representative patch (Figure 1) and the S_a_ value with the standard deviation (SD) of the implant in the study by Halldin et al. [27] are presented in Table 3. The fictitious Young’s modulus E_s_ of the bone surrounding a 3.5 mm (D_i_) implant with different values of the outer diameter (D_0_) is presented in Figure 5. The fictitious Young’s modulus was chosen to 33 MPa. A reasonable computing time was obtained with an acceleration of 600 m/s^2^ (resulting in a simulation time of 5 × 10^−5^ s), material density increase by a factor of 1,000 and a minimum element size of 1 µm. The results of the 2D analysis indicate that these settings do not seem to significantly affect the reaction force compared to simulation results using lower parameters values (P1, P2 and P3) (Figure 6). Hence these settings were used in the 3D simulations which resulted in a computing time of 24 h for each simulation. The 3D models were meshed with ANSYS^®^ default settings and a minimum element size of 1 µm resulting in meshes according to Table 4 and Figure 7. The interfacial shear strength of the three surfaces is presented in Figure 8a. The theoretical removal torques, according to Eq. (1), and the corresponding *in vivo* removal torques of Halldin et al. [27] are presented in Figure 8b. The ratio (*test/control*) of the simulated shear strength and the mean of the corresponding ratios of the *in vivo* study by Halldin et al. [27] for the surfaces are presented in Figure 8c.

**Table 3:** Surface characterization

	Parameter	CB-HF	CB-AT-1	FB-AT-1
Implant	S_a_	1.52 (SD 0.23)	1.49 (SD) 0.23	0.95 (SD 0.19)
Representative patch	S_a_ [μm]	1.48	1.63	0.91
	S_q_ [μm]	1.93	2.08	1.21
	Ssk [μm/μm]	0.18	−0.07	−0.44
	Sdq [μm/μm]	1.00	1.02	0.69

The surface roughness (S_a_ value) of the selected representative patch and the S_a_ value of the implant in the study by Halldin et al. [27].

**Table 4:** Mesh description

Surface	Number of nodes	Number of elements	Element type
CB-HF	378,131	2,025,934	Four-node tetrahedral element
CB-AT-1	356,030	1,888,789	Four-node tetrahedral element
FB-AT-1	314,164	1,662,405	Four-node tetrahedral element

Results of the mesh when using ANSYS® predefined meshing algorithm with a minimum element size of 1 µm and a defeaturing tolerance of 0.75 µm.

## Discussion

In the present study the theoretical interlocking capacity of three different implant surfaces with bone after different healing times was evaluated. The simulated ratios (test/control) seem to agree with those of the Halldin et al. [27] study for longer healing times (Figure 8). However, the simulated absolute removal torque is underestimated compared to the biological removal torque as found in Halldin et al. [27] (Figure 8b). There are many factors that may influence the simulated results: (1) model setup (2) representative mechanical properties of the interfacial bone during healing (3) selection of the representative patch (4) pressure at the bone-to-implant interface (5) bone-to-implant contact. In simulation it is essential to use a model that captures the essence of what is intended to be analyzed. However, a model will always be a simplification of reality. In this study a simplified model of the interface was developed to analyze the interlocking capacity of a rough surface with bone.

In the present 3D-model the circumferential structural displacement was neglected. The circumferential structural displacement would affect the angle of rotation but is not assumed to affect the interfacial shear strength. This simplification reduces the size of the model and thus the computing time. The 2D analyses were performed to investigate how the reaction force is affected by different simulation settings. A reduced element size was assumed to provide a higher accuracy but would have implied a longer computing time [29]. The results of the 2D analysis showed that a minimum element size of 1 µm could be used without negatively affecting the accuracy of the results. Furthermore to reduce computing time the simulation time was decreased by the use of increased acceleration. An increased simulated acceleration might affect the interfacial shear force. To reduce the inertia effects the simulation was performed with a constant acceleration of 600 m/s^2^, which ramped up the velocity from 0 to 30 mm/s during 5 × 10^−5^ s. To reduce the computing time even further the material density was increased in turn affecting the inertia and consequently the results. In the 2D analysis it was found that the material density could be increased by a factor of 1,000 without substantially affecting the interfacial shear strength.

The magnitude of the interfacial shear strength is affected by the mechanical properties of interfacial bone as well as the interface properties (i.e. friction) To the authors’ knowledge the coefficient of friction on micro level is unknown and the assumed coefficient of friction was based on a coefficient of friction between bone and titanium [28]. The coefficient of friction is assumed to affect the magnitude of the interfacial shear strength to the same extent between the simulations.

The knowledge about the mechanical properties of bone, including failure behavior, in the vicinity of an implant surface during healing is limited. Therefore, an estimation of the mechanical properties of bone was derived from the mineral content measurements of osteoid under mineralization in an osteon during healing by Fuchs [15] and the relationship between mineral content and material properties found by Currey [17]. Furthermore, it was assumed that the mineral content was 100% after 350 days of healing which corresponds to a Young’s modulus of 7.95 GPa [21]. To estimate the degree of mineralization during the first weeks of healing, extrapolation was made of the experimental data obtained by Currey [17]. Thus in the present study, Young’s modulus during healing was set in the range of 2–10 GPa. Other studies have found a variation in Young’s modulus during healing which could be caused by differences in experimental setup and choice of animals used [13, 23–25, 30]. The assumed mechanical properties used in this study might not reflect the true material properties during healing which in turn affect the absolute value of the removal torque. However, when the relative interfacial load bearing capacity of two surfaces is considered the actual material property during healing is assumed to have limited impact. Furthermore, the same bone material properties were used for all surfaces at coinciding healing times and a potential biological change in the mechanical properties caused by a specific surface topography was not considered. This might be an explanation of the consistency in ratios for longer healing times and the deviations during the early healing phase. This indicates that, for longer healing times the surface topography might be the main parameter influencing the interlocking capacity of an osseointegrated implant surface.

In this simulation the ultimate strain was used as a failure criterion based on available ultimate strain data. Principal strain failure can be used to represent brittle or ductile failure in materials [29]. Other material failure criteria, such as Mohr–Coulomb, Tsai–Wu and Hill, have been discussed for bone [31]. However to our knowledge the anisotropy and a suitable yield and failure criterion for the bone material during healing in the vicinity of the implant surface are unknown. When the failure criterion was reached in an element the element was removed and did no longer contribute to the interfacial shear strength. This might result in an underestimation of the simulated interfacial shear strength value. The magnitude of the reaction force depends on the surface topography of the selected patch. The standard deviation of the S_a_ value (Table 3) of the implants in the Halldin et al. [27] study, from which the representative patches were selected, indicates a variation. Even though the S_a_ value of the representative patch was selected to be in the range of the S_a_ value of the implant, the selected patch might not fully represent the surface topography with respect to load bearing capacity. When an implant is inserted it induces static strains in the bone that gradually decrease during healing [32, 33]. These initial residual stresses increase the removal torque value during the early healing period. The current simulations did not include the change of residual stresses during healing time. Therefore, the absolute removal torque value might be underestimated during the early healing phase. During osseintegration the biological processes start to integrate the implant with bone and thus increase the bone-to-implant contact and the removal torque over time. In this simulation, 40% bone-to-implant contact was assumed, for coinciding healing times, which might lead to an over- or underestimated simulated absolute removal torque, but is assumed to have a limited impact when analyzing the ratios.

## Conclusion

In this simulation the theoretical interfacial shear strength of the three surface topographies was simulated and compared to the results in the study by Halldin et al. [27]. The simulated ratios (test/control) seem to agree with those of the Halldin et al. [27] for longer healing times (Figure 8). Despite differences in the absolute removal torque values between simulations and the *in vivo* data, finite element analysis is a promising method, that can be used prior to *in vivo* study, to compare the theoretical load bearing capacities of the bone-to-implant interfaces especially for longer healing times. It was also concluded that the mechanical properties and the material model of bone in the vicinity of an implant affect the theoretical load bearing capacity and requires more research.

## Abbreviations

BIC: bone to implant contact
RTQ: removal torque.
